# Psychological Interventions for Reducing Distress in Patients with Cardiac Arrhythmias

**DOI:** 10.1007/s11886-025-02253-4

**Published:** 2025-06-25

**Authors:** Josefin Särnholm, Allison E. Gaffey, Meghan Reading Turchio, Angelo Biviano, Matthew M. Burg

**Affiliations:** 1 Department of Clinical Neuroscience, Division of Psychology, Karolinska Institutet, Nobels väg 9, Stockholm, 171 65 Sweden; 2 Center for Behavioral Cardiovascular Health, Columbia University Irving Medical Center, New York, NY USA; 3 Section of Cardiovascular Medicine, Department of Internal Medicine, Yale School of Medicine, New Haven, CT USA; 4 VA Connecticut Healthcare System, West Haven, CT USA; 5 Columbia University School of Nursing, New York, NY USA; 6 Division of Cardiology, Department of Medicine, Columbia University Vagelos College of Physicians and Surgeons, New York, NY USA; 7 Department of Anesthesiology, Yale School of Medicine, New Haven, CT USA

**Keywords:** Atrial fibrillation, Ventricular arrhythmias, Psychological distress, Anxiety, Depression, Cognitive behavioral therapy

## Abstract

**Purpose of Review:**

Cardiac arrhythmias, including atrial fibrillation (AF) and ventricular arrhythmias (VA), are associated with anxiety, depression, and poor quality of life (QoL). These and related aspects of psychological health significantly influence the clinical course and prognosis of arrhythmia patients, yet their integration into routine arrhythmia care remain limited. This focused review examines the use of psychological interventions, particularly cognitive behavioral therapy (CBT), in addressing psychological health in arrhythmia patients and discusses strategies for integrating these interventions into arrhythmia care.

**Recent Findings:**

CBT has demonstrated efficacy in reducing anxiety, depression, cardiac-related fears, and avoidance behaviors while improving QoL in arrhythmia patients.

**Summary:**

Further research is necessary to evaluate the effectiveness of these interventions in real-world care systems. Multidisciplinary care collaborations are essential for advancing the integration of psychological health within arrhythmia care. Integrating CBT for psychological health into arrhythmia care can improve clinical outcomes.

## Introduction

Cardiac arrhythmias are associated with distressing symptoms and impaired quality of life (QoL), with clinical outcomes ranging from benign episodes to life-threatening events [1, 2]. Substantial research shows that anxiety and depression are prevalent among patients with arrhythmias, and the conditions have a bidirectional influence on both QoL and arrhythmia outcomes. This association is particularly evident in patients with atrial fibrillation (AF), ventricular arrhythmias (VA), and those with implantable cardioverter defibrillators (ICDs) [3, 4]. Psychological health, which includes depression and anxiety, is increasingly recognized as a vital component of cardiovascular health and prognosis, as evidenced by a consensus statement from the American Heart Association (AHA) and other leading cardiology organizations [5–7]. Growing attention concerns the role of psychological health in overall arrhythmia care [2, 8–10], as current treatments do not sufficiently alleviate symptoms, leading to persistent distress for many patients [11, 12]. Consequently, there is a clinical need to integrate psychological care within the medical management of arrhythmias, as a conjunctive strategy to enhance overall patient outcomes.

This focused review explores the role of psychological health among patients with AF, VA, and also with ICDs, where psychological interventions have been studied more extensively. The paper also includes a review of current evidence for interventions targeting psychological distress in patients with AF, and VT, including ICD recipients and a discussion of strategies for integrating these interventions into comprehensive arrhythmia care.

### Psychological Health in Atrial Fibrillation

AF, the most common sustained arrhythmia, significantly increases the risk of stroke and heart failure.^1111^ Studies estimate that 25–50% of patients with AF experience elevated symptoms of anxiety and depression [3]. Patients with AF often experience markedly reduced QoL—not only compared to healthy individuals but also relative to those with other cardiac conditions, such as coronary heart disease [13]. Restoring and maintaining QoL in AF is a recognized treatment goal, alongside the traditional focus on stroke prevention and rate and rhythm control [9]. Depression is associated with AF recurrence after cardioversion [14] and anxiety has been associated with recurrence following catheter ablation [15]. Furthermore, each correlate with more severe self-reported AF symptoms and increased healthcare-seeking behaviors, irrespective of actual AF burden [16, 17]. Psychological symptoms and AF perpetuate one another in a cyclical fashion; psychological distress is often reported as an AF episode trigger [18], while living with AF can contribute to elevated anxiety, depression, and reduced QoL [19], as patients often limit activities due to fear and anxiety of triggering AF symptoms [20]. This pattern of fear-based avoidance is clinically relevant, as psychological distress and symptom preoccupation are shown to worsen self-rated symptom severity and AF-related disability, independent of arrhythmia burden [21]. These findings show the importance of addressing psychological health in AF care to improve psychological health and overall patient outcomes.

### Psychological Health in Ventricular Arrhythmias and ICD Recipients

VA, including life-threatening forms such as ventricular tachycardia and ventricular fibrillation, pose substantial health risks and often necessitate ICDs to prevent sudden cardiac arrest [22]. A recent meta-analysis of 39,954 ICD recipients reported prevalence rates of anxiety, depression, and posttraumatic stress at 23%, 15%, and 12% respectively, more than 12 months after implantation [23]. Rates of anxiety exceed that of depression, with fear of arrhythmia recurrence or ICD-related events (e.g., shock) postulated as key drivers of poor psychological health outcomes, while psychological distress was particularly elevated among patients who experienced ICD shocks [23]. Patients with ICDs typically report lower physical and mental quality of life compared to age- and sex-matched population norms [24]. In a sample of 2,658 ICD recipients, anxiety, depression, a history of ICD shocks, high ICD-related concerns, and female sex, were all associated with lower QoL [25]. Factors associated with psychological health, particularly depression and anxiety, are consistently shown to increase VA recurrence and contribute to poorer cardiac prognosis and heightened mortality risk [26–29]. For instance, a recent prospective cohort study (*n* = 399) found that patients with depression at the time of ICD implantation had a 2x greater risk of mortality over seven years of follow-up (95% CI:1.44–3.05, *p* < 0.001).^4^

In summary, patients with cardiac arrhythmias often experience poor psychological health, including cardiac-related distress, anxiety, depression, and reduced QoL. This compromised psychological health can also influence the course of their arrhythmia, contributing to recurrence, increased healthcare utilization, a poorer cardiac prognosis, and a higher mortality risk.

### Arrhythmia-Specific Psychological Health: Cardiac-Related Distress and Avoidance Behavior

In addition to comorbid general anxiety and depression, disease-specific aspects of psychological health, such as cardiac-related fear, hypervigilance (i.e., heightened and excessive attention), and avoidance behavior–together referred to as symptom preoccupation or cardiac anxiety–are particularly relevant in patients with arrhythmias [30–32]. Hypervigilance to cardiac-related sensations, fear of symptom recurrence or of ICD shock perpetuate avoidance behavior (e.g., of physical activities or other perceived triggers), impairing QoL and daily functioning [30–32]. In AF, it is well established that symptom preoccupation and psychological distress contribute to worse self-rated symptom severity and AF-related disability [21, 31, 33]. Notably, neither QoL impairment, symptom frequency, nor symptom duration are correlated with overall AF burden [1, 34], suggesting that factors beyond the arrhythmia burden play a significant role. Disease-specific behavioral patterns of clinical interest include fear of triggering or experiencing AF-related symptoms, hypervigilance to cardiac and AF-related symptoms, and avoidance of social or physical activities or situations that may increase heart rate [35–37]. Similarly, in patients with VA, ICD-specific anxiety or fear of ICD shocks is prevalent and often leads to excessive avoidance and functional impairment [38–40]. It is worth noting that there is a weak correlation between shock experience and shock anxiety; many ICD patients experience significant distress despite not receiving any shocks [31, 41]. In both AF and ICD patients, hypervigilance and heightened awareness of internal sensations, such as normal changes in heart rate or rhythm, contribute to the misapprehension of normal cardiac sensations and a perceived risk of recurrent symptoms or shocks [31, 40, 42]. Through interoceptive fear conditioning, cardiac-related symptoms and bodily sensations become repeatedly associated with fear or negative emotions. This learned association can cause these sensations to automatically trigger anxiety and autonomic arousal—such as increased heart rate and palpitations—even in the absence of an actual threat. This heightened physiological response may, in turn, contribute to or exacerbate arrhythmias [43, 44]. Anxiety is also associated with autonomic nervous system activation, a pro-inflammatory state, and stress triggers, all of which promote arrhythmia and may aggravate symptoms [45]. 

Lifestyle factors (e.g., engaging in regular physical activity) are recognized as the “fourth pillar” in AF management, alongside rhythm control, rate control, and stroke prevention, as optimal lifestyle can significantly reduce symptom burden and AF recurrence, and improve QoL [46, 47]. Indeed, regular physical activity, in particular, has shown profound benefits in arrhythmia management [46, 47]. Yet, anxiety, especially cardiac-specific anxiety, may create substantial barriers in adherence to lifestyle interventions by amplifying symptom perception and fostering avoidance behaviors [48]. Patients with arrhythmias may avoid physical activity to prevent the sensations associated with an increased heart rate, as these can trigger fear of inducing an arrhythmic episode. They may also worry that the heightened adrenergic state during exercise could provoke or worsen their arrhythmia. This avoidance behavior is thereby ‘reinforced’ by the temporary reduction in anxiety that it provides and perpetuates the fear response to cardiac-related sensations and symptoms [38, 42]. Anxiety over recurrent symptoms, fear associated with ICD shock, and cardiac-related avoidance behaviors may interfere with daily activities and a healthy overall lifestyle (e.g., engaging in regular physical activity) [49]. Of these factors, avoidance behavior has been specifically associated with depression in patients with arrhythmias [38]. Importantly, the physical activity that patients avoid is one of the key behaviors needed to improve cardiac health, and avoidance perpetuates deconditioning. These insights highlight the importance of addressing disease-specific psychological health, including cardiac-related fear and avoidance behaviors in arrhythmia management.

### Psychological Interventions in Arrhythmias

A 2022 Cochrane review of 21 psychological intervention studies (*n* = 2,591), specifically focusing on cognitive behavioral therapy (CBT) for cardiac populations, showed the approach was effective in reducing depression and anxiety and improving QoL, although these interventions had no significant effect on mortality or major cardiovascular events [50]. While the review included studies with coronary artery disease and heart failure patients, the authors found no studies had specifically focused on patients with AF and recommended further randomized controlled trials (RCTs) of psychological interventions for AF and other arrhythmias [50]. 

CBT is a first-line, effective treatment for anxiety and depression, demonstrating equivalent treatment effects whether delivered face-to-face or online, while also offering the advantage of scalability [51]. Exposure-based CBT is particularly effective for anxiety-related issues, as it disrupts the cycle of fear and avoidance through gradual experience with feared stimuli and situations [52]. 

#### Psychological Interventions Targeting Distress in Patients with Atrial Fibrillation

An AF-specific, exposure-based CBT (AF-CBT) intervention targeting cardiac-related fear and AF-related avoidance behaviors was evaluated through two pilot studies and an RCT, published in 2023 [35–37]. The RCT, included 127 patients with symptomatic paroxysmal AF who were randomly assigned to receive either AF-CBT or standardized AF education [36]. The 10-week, therapist-led intervention was delivered via the internet and included interoceptive exposure to AF-like physical sensations, such as increased heart rate induced by running on the spot, to reduce fear of AF-like symptoms. This strategy was supplemented with gradual exposure to feared or avoided situations. The RCT demonstrated large and significant improvements in AF-specific QoL, with a mean increase of 15 points on the Atrial Fibrillation Effect on Quality of Life (AFEQT) summary score (95% CI: 10.1–19.8; *P* < 0.001). Furthermore, AF-CBT was associated with a 56% reduction in AF-related healthcare utilization (95% CI: 22–90; *P* = 0.025), while ECG-measured AF burden remained unchanged. Notably, AF-specific QoL improvements in the intervention group were comparable to those reported in ablation trials and sustained at 12-month follow-up [36]. Mediation analyses identified reductions in cardiac-related fear, hypervigilance, and avoidance behaviors as key mechanisms driving improvements in AF-related disability and self-reported symptom severity [37, 53]. A secondary analysis also showed improved self-reported sleep quality compared to the control group (*P* = 0.032) but no significant changes in physical activity or heart rate variability [54]. 

Another behavioral intervention for atrial fibrillation (AF) included dyadic mindfulness-based CBT for patients with AF and their spouses [55]. The RCT included 111 patients and their spouses, randomized to mindfulness-based CBT or usual care (UC). The intervention, delivered in three 2.5-hour group sessions over nine weeks, resulted in significantly greater health-related QoL (HRQoL) improvements at 12 months for the CBT group. Other RCTs testing face-to-face CBT, adapted from both depression and anxiety treatments, have demonstrated improvements in QoL, depression, and anxiety [56, 57]. One such trial including 90 patients with AF compared 12 face-to-face CBT sessions (*n* = 45) with UC (*n* = 45). This intervention, which included cognitive restructuring and behavioral activation, significantly improved mental HRQoL (*P* < 0.001), depression (*P* = 0.002) and illness perception (*P* < 0.001).^50^ Similarly, an RCT of 90 paroxysmal patients with AF evaluated a 10-week CBT program targeting anxiety. At 6-month follow-up, the CBT group showed significant improvements in mental HRQoL (*p* < 0.001), anxiety (*p* < 0.001), illness perception (*p* < 0.001), and AF severity (*p* = 0.001) compared with standard care [51]. 

#### Interventions to Improve Psychological Health for Patients with Ventricular Arrhythmias and ICDs

A range of interventions to improve psychological health have been evaluated in randomized trials for ICD patients, with promising outcomes. A recent systematic review and meta-analysis of 675 ICD patients demonstrated that CBT effectively reduces depression and anxiety (95% CI: -1.10 to -0.30; *P* < 0.001) compared to usual care, underscoring its efficacy as a targeted psychological intervention [58]. These findings are reaffirmed by Berg et al. (2019) [59]who randomized 88 ICD patients to either anxiety-targeted CBT (e.g., psychoeducation, restructuring of negative automatic thoughts, exposure) or usual care (UC). The investigators found significant reductions in anxiety for the CBT group (Cohen’s d = -0.86; *P* < 0.0001), indicating a strong clinical effect. Additionally, in a study of 193 patients randomized to either eight weeks of ICD-specific CBT—focused on facilitating adjustment to the ICD and addressing anxiety and depressive reactions to ICD shocks—or UC, CBT demonstrated significantly greater improvements, including reductions in symptoms of posttraumatic stress, depressive symptoms, and improved HRQOL compared with UC [60]. The effects observed in the CBT group were maintained over a 12-month follow-up period [60]. Moreover, a randomized trial of 103 ICD patients compared the effects of a 10-week cognitive behavioral stress management (CBSM) program to patient education on mood and cardiovascular responses to stress. CBSM resulted in moderate reductions in anxiety (*P* = 0.010), anger (*P* = 0.020) and perceived stress (*P* = 0.037), but these effects were not sustained at six months, and no significant effects on heart rate variability, hemodynamic stress responses, or arrhythmias were observed [61]. 

Digital interventions have gained increasing attention as a way to scale and bridge treatment gaps in healthcare. In a study by Schulz et al., a six-week, web-based psychosocial intervention for ICD patients with elevated psychological distress was evaluated. The design involved 118 participants randomized to the intervention–CBT-based self-help interventions, a virtual self-help group, and psychologist support–or UC. At the 1-year follow-up, the intervention group showed significant improvements in depression (*P* = 0.02), anxiety (*P* = 0.03), and social support (*P* = 0.047). While effective and scalable, the study highlighted limitations like broad content, suggesting future efforts should focus on tailored, ICD-specific interventions [62]. 

CBT for other types of VA remains understudied. However, a recent pilot study adapted the digital exposure-based AF-CBT [36] protocol for patients with symptomatic idiopathic premature ventricular contractions (PVCs). PVC-specific CBT was delivered over eight weeks by a licensed psychologist via videoconference, supplemented with online text-based information and homework assignments. The findings suggest that the exposure-based approach is feasible, acceptable, and shows promise for improving PVC-specific quality of life, reducing symptom preoccupation, and lowering self-reported PVC symptoms [63]. 

### Integration of Psychological Interventions

**Fig. 1:**
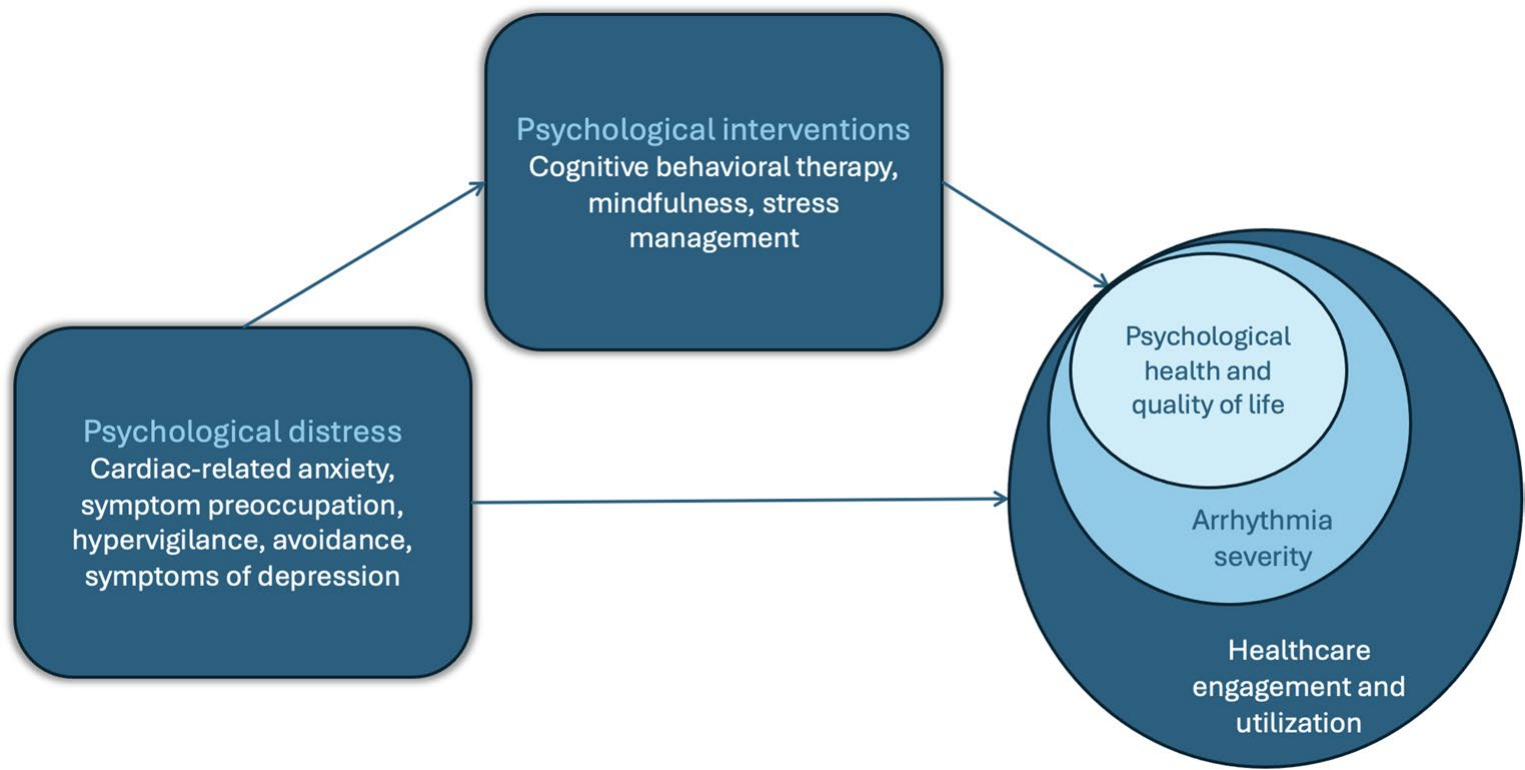
Integration of psychological distress interventions in arrhythmia care Psychological distress—including cardiac-related anxiety, symptom preoccupation, hypervigilance, avoidance, and depressive symptoms—can negatively impact quality of life, perceived arrhythmia severity, and healthcare engagement. Psychological interventions such as cognitive behavioral therapy, mindfulness, and stress management aim to reduce distress and improve psychological health. These interventions may enhance outcomes across multiple domains relevant to arrhythmia care. **Note.** The arrow from *Psychological distress* to *Psychological Interventions* reflects a proposed clinical pathway in which elevated distress may lead to identification, referral, or engagement in treatment

Altogether, psychological interventions, such as CBT, have demonstrated significant benefits for patients with cardiac arrhythmias, including alleviating anxiety and depression, addressing cardiac-related fears and avoidance behaviors, and improving QoL. Digital CBT shows promise as a scalable and accessible solution, with tailored interventions targeting disease-specific behavioral patterns proving particularly effective. These findings highlight the potential of integrating attention to psychological health (e.g., through use of CBT) into overall arrhythmia care (See Fig. 1). Despite clear evidence of anxiety and depression as independent risk factors for poor cardiac outcomes, and substantial evidence supporting safe and effective treatment [64], the symptoms of these conditions remain undetected and untreated for the majority of arrhythmia patients with psychological distress, and the implementation of psychological interventions continues to be inconsistent [6]. While clinical guidelines recommend assessing and treating psychological distress in ICD patients [12], parallel recommendations have yet to be integrated into clinical guidelines for AF management. Below, potential clinical implications and future directions are discussed.

#### Screening for Psychological Distress

Patient-reported outcome measures are increasingly recognized as valuable tools in clinical research and practice [65–67]. These measures provide direct insights into patient experiences, including their symptoms, which are often overlooked in traditional clinical assessments. They can help identify patients with elevated psychological distress, plan targeted interventions, assist healthcare providers in determining when to refer patients for psychological intervention, evaluate treatment efficacy, and compare the effectiveness of different interventions [65–67]. Despite frequent use of patient-reported outcome measures in clinical trials, the measures are underused in clinical practice [68]. 

#### Integrating Patient-Reported Outcome Measures in Clinical Care

There are increasing, promising efforts to better incorporate patient-reported outcome measures into cardiac care. A multidisciplinary electrophysiology-psychology clinic successfully integrated general and cardiac-specific self-reported outcome measures for patients with VAs [69]. Anxiety and depression symptoms were not correlated with traditional clinical markers (e.g., defibrillator shocks, ejection fraction), but were frequently identified through routine use of the Patient-Reported Outcomes Measurement Information System (PROMIS) questions. Key concerns included returning to physical activity, resuming normal sexual activity, and maintaining employment [69]. Cardiac-specific assessment can capture the unique sources of fear, avoidance, and functional impairment specific to each patient and the authors recommend routine use of these measurements at initial and follow-up visits to monitor distress and guide referrals, underscoring their value in enhancing patient-centered care [2, 69]. Similarly in AF, these assessments serve as essential tools for evaluating health status, supporting shared decision-making, tailoring treatment intensity, and offering prognostic insights to enhance clinical care [70]. 

More specifically, screening for psychological distress and AF-specific fears or impairments could be implemented at the time of diagnosis as part of pre-ablation therapy preparation, or during follow-up care. For example, arrhythmia-specific or cardiac-specific patient-reported outcome measures (e.g., the Cardiac Anxiety Questionnaire [71], Atrial Fibrillation Effect on Quality of Life Questionnaire [72], Florida Patient Acceptance Survey and Florida Shock Anxiety Scale [41, 73]), provide disease-specific assessments. These tools are particularly valuable for evaluating cardiac-related fear and avoidance behaviors, and offer insights that are essential for understanding the psychological impact of arrhythmias. The assessments would ideally be integrated into cardiovascular care pathways as a first step before referring to a clinical psychologist. In a next step before treatment, the use of disease-specific measures with structured clinical assessments conducted by psychologists is essential for addressing behaviors that drive disability, such as cardiac-specific fear or avoidance behaviors, directly associated with the arrhythmia. Including a structured psychological assessment ensures that therapy is accurately targeted to address the primary clinical problem. Idiographic assessment with behavioral analysis further helps identify the specific fears, avoidance patterns, and functional impairments in each patient. While arrhythmia patients with and without ICDs may share a fear of unpredictable symptoms or shocks, the source of distress often differs (e.g., fear of AF recurrence vs. anxiety about receiving electrical shocks). This underscores the need for tailored psychological interventions grounded in rationales that patients can understand and relate to, and that cardiovascular medicine providers can recommend to complement other aspects of arrhythmia care.

#### Incorporating Psychologists in Arrhythmia Management

Integrating health psychology into cardiac electrophysiology is essential. Clinical psychologists should serve as key members of cardiovascular care teams, providing routine clinical services, supervising health psychology trainees, and contributing to the education of cardiology fellows [74–76]. Multidisciplinary team approaches, using evidence-based practices and guidelines, have shown positive effects on patient outcomes in cardiovascular care [77]. For example, collaborative care models tested in CVD patients could be adapted for arrhythmia care, where specialty providers and behavioral health professionals (e.g., cardiologists, psychologists, and nurses) work together. These models have demonstrated improvements in psychological health, QoL, self-management, cardiovascular markers, and cardiac function, with potential long-term benefits on hospitalization, readmission, and mortality rates [78]. 

To implement these interventions effectively, psychologists should receive foundational training in cardiovascular diseases and close collaboration with medical teams. Successful training models highlight the importance of educating health psychologists alongside cardiac providers, equipping them with the specialized skills necessary to address the distinct challenges of cardiac care [74, 75]. For example, in the delivery of arrhythmia-specific exposure-based digital CBT, pre-enrollment cardiac and psychological assessments were conducted, and psychologists had access to on-demand consultation with cardiac nurses and cardiologists during treatment to address emergent cardiac symptoms [36, 37, 63]. This approach ensures patient safety, promotes engagement with exposure strategies and physical activity, and fosters integration and collaboration between psychological and medical teams. Despite their efficacy, exposure-based approaches remain underutilized in cardiac care, often due to safety concerns or lack of available trained professionals [79], which underscores the importance of multidisciplinary collaboration in delivering these interventions effectively.

Integrating psychologically-informed education in arrhythmia care is an important step to address disease-specific misconceptions and to educate patients in ways that normalize common behavioral responses while identifying maladaptive patterns, such as avoidance behaviors, which can exacerbate distress or hinder treatment adherence. Brief psychoeducational interventions, delivered both pre and post ICD implantation, have significantly reduced depression, anxiety, and healthcare utilization [80, 81] Similarly, educational interventions in patients with AF have demonstrated improved outcomes, highlighting the importance of preparatory and rehabilitative education for these populations [82]. Psychologically-informed education could be provided before interventions such as ablation or ICD implantation, at the time of an AF diagnosis, or during follow-up care. Standardized psychologically-informed education could also be delivered by medical staff and serve as a valuable resource for educating caregivers. As part of a stepped care approach, patient education can serve as an initial, low-intensity intervention, providing foundational knowledge and psychological support, with more intensive interventions offered based on individual needs and levels of distress.

In summary, systematic screening for psychological distress and the use of patient-reported outcome measures in patients with arrhythmias remain inconsistent, leaving many psychological needs unaddressed. Adopting an integrative approach that incorporates psychologists into cardiac care teams is essential. Based on the growing evidence, this approach should emphasize routine screening for psychological distress, psychologically-informed patient education, psychologist-led assessments, and individualized therapy to promote psychological well-being and improve clinical outcomes for patients with AF and ICDs.

## Conclusions and Future Directions

Psychological health significantly influences the clinical course in patients with arrhythmias. Anxiety, depression, cardiac-related fear, and avoidance behaviors are common in these populations and have a profound impact on QoL, adherence to health behaviors, and treatment outcomes. Psychological interventions, especially CBT, have demonstrated considerable benefits among patients with arrhythmia, including improved mental health, reduced avoidance behaviors, enhanced QoL, and decreased healthcare utilization.

Despite these demonstrated advantages, the integration of psychological assessment and care into routine arrhythmia management remains inconsistent, even rare. To bridge this gap, future efforts should prioritize the implementation of routine and recurrent psychological screening using validated tools and, structured clinical assessments to identify at-risk patients. A multidisciplinary approach that combines cardiovascular and psychological health management is vital, with psychologists playing a supportive role in cardiovascular care teams. While promising RCTs provide a strong evidence base, further research is necessary to evaluate the effectiveness of these interventions in real-world care systems, measuring their generalizability to diverse arrhythmia populations and healthcare settings. Future studies should also investigate mechanisms of treatment response, including the potential mediating role of avoidance behavior. Longitudinal and process-oriented research designs may help clarify the temporal dynamics of psychological change and support the development of more targeted interventions. Implementing these strategies, alongside validated screening tools and structured psychological assessment, and leveraging psychological interventions as a conjunctive treatment, healthcare systems can enhance patient outcomes, reduce resource burden, and establish a more comprehensive standard of care for patients with arrhythmias.

## Key References

Särnholm J, Skúladóttir H, Rück C, et al. Cognitive Behavioral Therapy Improves Quality of Life in Patients With Symptomatic Paroxysmal Atrial Fibrillation. *J Am Coll Cardiol*. 2023;82(1):46–56. doi:10.1016/j.jacc.2023.04.044.

**Exposure-based digital cognitive behavioral therapy (CBT) targeting cardiac-related fear and avoidance behaviors significantly improves quality of life in patients with symptomatic paroxysmal atrial fibrillation.**

Li Z, Liu Y, Wang J, Zhang C, Liu Y. Effectiveness of cognitive behavioral therapy on mood symptoms in patients with implantable cardioverter defibrillator: A systematic review and meta-analysis. *Complement Ther Clin Pr*. 2022;47:101570. doi:10.1016/j.ctcp.2022.101570.

**This systematic review and meta-analysis found that cognitive behavioral therapy (CBT) effectively reduces psychological distress**,** including anxiety and depression**,** in patients with implantable cardioverter defibrillators. The findings support CBT as a valuable intervention for improving psychological health in this patient population.**

Burg MM, Stewart JC, Gaffey AE, Särnholm J, Vela AM, Kovacs RJ. Behavioral Medicine Can Tackle Cardiovascular Disease Risk and Burden: A Call for New Priorities. *Polic Insights Behav Brain Sci*. Published online 2025. doi:10.1177/23727322241306071.

**This article highlights the role of behavioral medicine**,** including psychological interventions**,** in reducing cardiovascular disease risk and burden**,** advocating for its integration into clinical care and public health. It calls for a shift in priorities to advance research**,** policy**,** and interdisciplinary collaboration in cardiovascular health.**

## Data Availability

No datasets were generated or analysed during the current study.
